# Sociodemographic and Medical Characteristics of Women Applied for Emergency Contraception—A Retrospective Observational Study

**DOI:** 10.3390/jcm13061673

**Published:** 2024-03-14

**Authors:** Richárd Tóth, Lotti Lőczi, Marianna Török, Attila Keszthelyi, Gergő Leipold, Nándor Ács, Szabolcs Várbíró, Márton Keszthelyi, Balázs Lintner

**Affiliations:** 1 Department of Obstetrics and Gynecology, Semmelweis University, 1082 Budapest, Hungary; toth.richard@semmelweis.hu (R.T.); keszthelyi.lotti.lucia@semmelweis.hu (L.L.); leipold.gergo@semmelweis.hu (G.L.); acs.nandor@med.semmelweis-univ.hu (N.Á.); varbiro.szabolcs@med.semmelweis-univ.hu (S.V.); keszthelyi.marton@semmelweis.hu (M.K.); lintnerster@gmail.com (B.L.); 2 Workgroup of Research Management, Doctoral School, Semmelweis University, 1085 Budapest, Hungary; 3 Department of Urology, Semmelweis University, 1082 Budapest, Hungary; attilakeszthelyi@hotmail.com; 4 Department of Obstetrics and Gynecology, University of Szeged, 6725 Szeged, Hungary

**Keywords:** emergency pill, lifestyle factors, reproductive health awareness, parenthood, age

## Abstract

**Background**: Lifestyle factors significantly impact overall health. Our aim was to assess reproductive health awareness among patients who applied for emergency contraceptive pills. **Methods**: This present retrospective observational study between July 2021 and September 2021 is embedded in the MEEC (Motivation and Epidemiology of Emergency Contraceptive Pill) based on the study cohort of a Hungarian data bank containing follow-up data of 447 women who applied for EC telemedicine consultation. Collected data: age, history of previous pregnancy, lifestyle factors like smoking, alcohol consumption, sexual characteristics: partner consistency and protection during intercourse, cervical cancer screening within the past 2 years, previous HPV screening, and the preference for future contraceptive methods. The investigation also compiled accurate data on intercourse (elapsed time to request a medical consultation). Lifestyle factors were scored. **Results**: The more health-conscious patients were quicker to report for a post-event pill. Earlier pregnancies and older age were associated with greater reproductive health awareness. **Conclusions**: Reproductive health awareness is increased by previous pregnancies and older age. More health-conscious women consult a doctor earlier, which can reduce the chance of various health damage. Our study emphasizes the significance of lifestyle factor influence on reproductive health decisions.

## 1. Introduction

Unintended pregnancies remain a persistent global public health challenge, underscoring the importance of accessible and effective emergency contraceptive options [1]. Emergency contraceptive pills (ECPs) provide a prompt and effective solution for averting unintended pregnancies; however, these pills are available by prescription only in Hungary [2]. The utilization of ECPs is correlated with a heightened level of awareness regarding fertility [3]. Motivators may vary, but the use of ECPs is influenced by various factors, presenting an interesting area of research [4].

Despite these challenges, it is noteworthy that approximately 61% of unwanted pregnancies are resolved through abortion [5]. Within the context of unintended pregnancies, women may choose abortion for various reasons rooted in their unique life circumstances. Abortion rates per 1000 women in Europe between the ages of 15 and 49 (44 in certain countries) and the abortion ratio per 1000 births also differ significantly [6].

Despite some progress through education, unintended pregnancy rates persist as a significant public health challenge, underscoring the importance of accessible and effective emergency contraceptive options [1]. Contraceptive methods have been thoroughly established in terms of their safety and efficacy [7]. Emergency contraceptive pills (ECPs) provide a prompt and effective solution for averting unintended pregnancies. ECP options include Levonorgestrel as a progestin-only pill, commonly taken as a single 1.5 mg dose or as two doses of 0.75 mg each, 12 h apart. Another choice is Ulipristal acetate, a progesterone receptor modulator, effective up to 120 h (5 days) after unprotected intercourse [8]. These pills are available by prescription only in Hungary [2]. Other options for emergency contraception are the Yuzpe method and IUDs. Two doses of a combination estrogen/progestin oral contraceptive—100 µg ethinyl estradiol and 1 mg dl-norgestrel, or 0.5 mg levonorgestrel—taken 12 h apart constitute the Yuzpe method. The Yuzpe method is effective within 3 days of the event [9]. The use of IUDs as emergency contraception may also be an alternative solution. They are effective within 5 days of the event [10,11].

Lifestyle includes daily behaviors and routines, professional commitments, recreational habits, and dietary patterns [12]. In recent years, there has been a heightened focus on the role of lifestyle as a determinant of health. The World Health Organization (WHO) underscores this significance, attributing a noteworthy 60% of factors influencing individual health and quality of life to the intricacies of lifestyle choices [13]. The impact of lifestyle choices extends across a broad spectrum of health issues, encompassing metabolic aberrations, musculoskeletal pathology, cardiovascular disorders, hypertension, obesity, and interpersonal violence, thereby underscoring the intricate and multifaceted nature of these choices. Awareness of the deleterious effects of smoking and drinking is widespread, yet individual choices in these areas may be intricately shaped by diverse social, cultural, and personal influences, irrespective of the potential health hazards [14,15]. Smoking and excessive alcohol consumption are detrimental to both general health and reproductive health [16], contributing to decreased fertility, adverse pregnancy outcomes, and long-term morbidity in offspring [17]. Additionally, smoking is known to increase the risk of infertility, and cervical cancer [18,19], while excessive alcohol consumption has been linked to fertility issues by disrupting hormonal balance in both genders and impairing reproductive functions. Both tobacco use and excessive alcohol consumption can have adverse effects on assisted reproductive technologies (ARTs) outcomes [20].

Over the past few decades, there has been a remarkable and discernible upswing in women’s enthusiasm for gaining a comprehensive understanding of and actively monitoring their menstrual and reproductive cycles [21,22]. Notably, this trend aligns with the explosive growth of mobile health applications, surpassing hundreds of applications dedicated to cycle tracking in the last years [22].

The increasing awareness of sexual health considerations, which encompasses facets such as human papillomavirus (HPV) and cervical screening, as well as the meticulous tracking of ovulation, further serves to underscore the contemporary emphasis placed on fertility awareness.

We aim to assess reproductive health awareness among women using emergency contraceptive pills. The aim of this study was to see if healthier patients seek medical advice earlier. Also, to investigate what factors influence reproductive health awareness among women using ECP.

## 2. Material and Methods

### 2.1. Patients

This retrospective observational study is part of the MEEC (Motivation and Epidemiology of Emergency Contraceptive Pill) initiative, utilizing data from a Hungarian database that includes follow-up information for 447 women [4]. From July 2021 to September 2021, a total of 447 individuals enrolled on the telemedicine consultation platform ‘https://esemenyutan.hu/’ (accessed on 1 February 2021), accessible to Hungarian health insurance holders seeking emergency contraceptive prescriptions following consultations with gynecologists. The service included a prompt consultation and prescription of the medication within an hour. During the consultations, patients were required to respond to a standardized set of questions, aiming to investigate their sexual behaviors and lifestyle.

### 2.2. Characteristics

This analysis involved a comprehensive examination of patient records, exploring a range of factors. These comprised sociodemographic details such as age (determined by subtracting the date of birth from the consultation date), prior pregnancy history, and lifestyle elements like smoking, alcohol use, and sexual behaviors, including partner consistency and protection during intercourse. Furthermore, health-related information, such as cervical cancer screening in the preceding two years, past HPV screening, and preferences for future contraceptive methods, was taken into consideration. The time elapsed since the sexual activity was also calculated.

The study was approved by the Institutional Review Board of Semmelweis University (SE RKEB: 125/2022).

### 2.3. Reproductive Awareness Score

The collected data underwent rigorous quality control procedures, including the elimination of repeated consultations (retaining only the first visits) and the rectification of data entry errors.

The scoring was based on the participants’ lifestyle decisions, reproductive health practices, and their inclinations toward future contraceptive choices.

The assessment of scoring involved a comprehensive analysis of various lifestyle factors, including smoking and alcohol consumption patterns. The study delved into participants’ relationship statuses, examining whether they were single, in a committed relationship, or married. Participants’ engagement with preventive health measures were assessed such as cervical cancer screening history within a two-year period and history of prior human papillomavirus screening. The assessment also took into account the ovulation status of the participants, exploring a key aspect of reproductive health. Further, the study scrutinized contraceptive practices during intercourse, including the use of condoms or withdrawal methods. Lastly, the study explored participants’ preferences regarding future contraceptive methods, specifically focusing on their inclination towards oral contraceptives or intrauterine devices (IUDs).

The following table (Table 1) shows the scores that participants could receive: evaluated based on their smoking and alcohol consumption patterns, relationship status, recent cervical cancer screening history within a two-year period, ovulation status, contraceptive use during intercourse (condom or withdrawal), previous human papillomavirus screening, and their preferences regarding future use of oral contraceptives or intrauterine devices (IUDs). Scores could range from 0 to 13, and the more points someone received, the more health-conscious they were.

### 2.4. Statistical Analysis

The Shapiro–Wilk test was used to test the normality of continuous variables. The Mann–Whitney test was used to analyze the relationship between awareness score and history of pregnancies. Pearson correlation was performed to assess the correlation between the awareness score and time or age. Statistical significance was set at *p* < 0.05. Prism9 GraphPad (ver. 8. GraphPad Software, Inc., San Diego, CA, USA) software was used for data management and analysis, and for creating figures.

## 3. Results

### 3.1. Patient Characteristics

In our study, Table 2 shows the distribution of patients in different health categories such as smoking, alcohol consumption, partner consistency, pap smear in the last 2 years, ovulation awareness, protection used, previous HPV screening, and further contraception desired.

**Figure 1:**
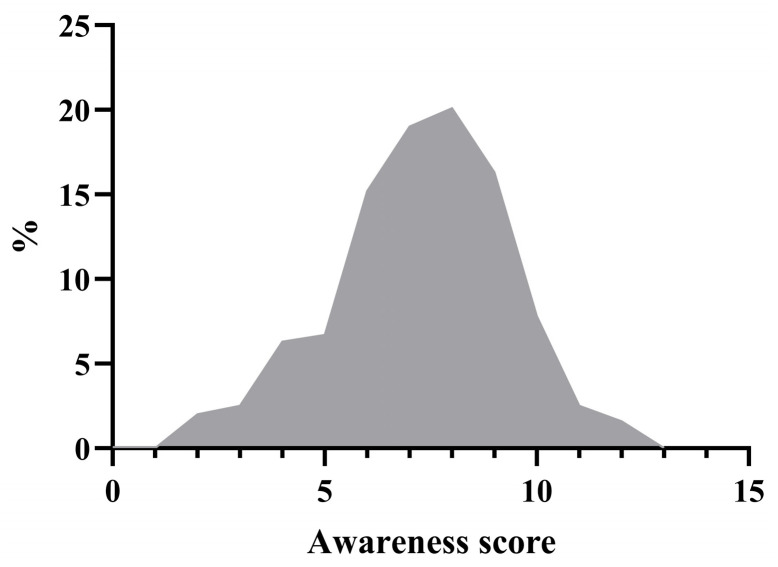
Reproductive health awareness score distribution.

Figure 1 shows in detail the distribution of the sample according to the reproductive health awareness scores obtained. The maximum score for the health categories was 13, which was not reached by any of the participants. The lowest number of points achieved was 2. It can be seen that the calculated scores are distributed according to a Gaussian curve, with the highest and lowest scores being obtained by a few, 1–2%, while the average score of 7 or 8 was obtained by 19 and 20% of the participants, respectively.

### 3.2. Reproductive Health Awareness Score and Elapsed Time

**Figure 2:**
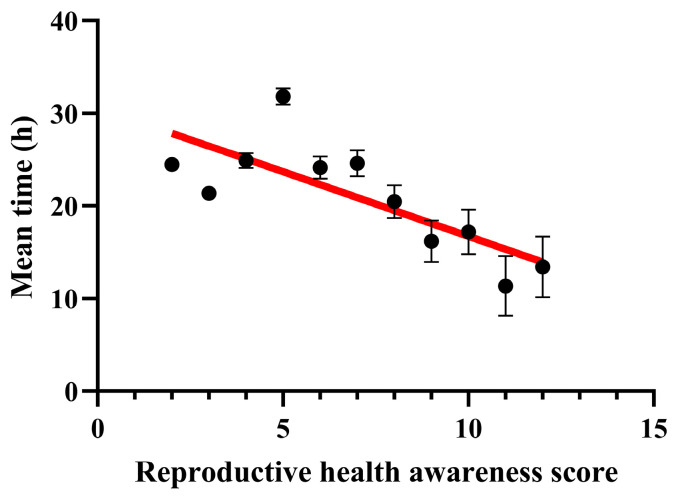
Mean time and reproductive health awareness score correlation. The time to call and the awareness score show a significant negative association. Data are presented as mean + SEM. Pearson correlation: r = −0.7755; R^2^ = 0.6014; *p* value = 0.005.

Linear regression analysis showed that the average time elapsed before requesting a medical consultation was inversely correlated with the reproductive awareness score. The more health-conscious women were, the faster they made a phone call (Figure 2).

### 3.3. Reproductive Health Awareness Score and Previous Pregnancy and Age

**Figure 3:**
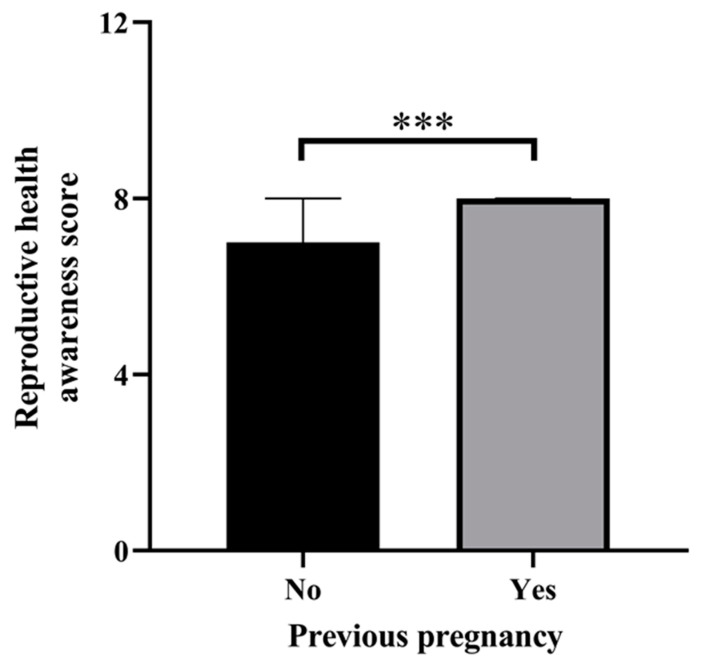
Previous pregnancy and reproductive health awareness score. The awareness score is significantly higher for women who have been pregnant before. Data are presented as median with 95% confidence interval confidence interval. Mann–Whitney test, *** *p* = 0.0007.

Women who had previously been pregnant had a significantly higher number of awareness points compared to those who had not been pregnant (Figure 3).

**Figure 4:**
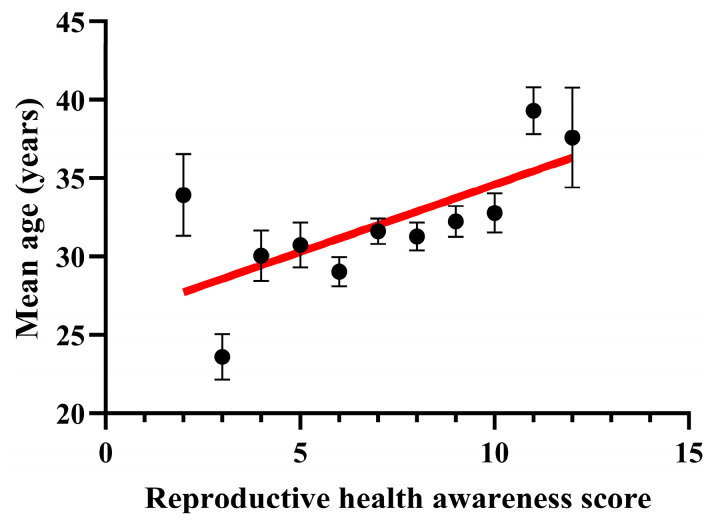
Age and reproductive health awareness score. With age, the awareness score showed a positive correlation. Data are presented as mean + SEM. Pearson correlation: r = 0.6823; R^2^ = 0.4655; *p* value = 0.0207.

Furthermore, the reproductive health awareness score increased significantly with age (Figure 4).

## 4. Discussion

In the present study, we demonstrated that reproductive health awareness is significantly influenced by factors such as previous pregnancies and age. The key findings can be summarized as follows: (1) patients who were more health-conscious sought medical advice more promptly; (2) women with a history of previous pregnancies exhibited higher levels of health consciousness; (3) reproductive health awareness showed an increase with age.

Our findings underscore that individuals with an elevated sense of health consciousness exhibit a noticeable tendency to promptly seek medical advice. This indicates a proactive and well-informed approach to healthcare, reflecting a prioritization and attunement to their overall well-being.

Our study demonstrates the association between reproductive health awareness and women who have undergone previous pregnancies. The findings underscore that women with a history of prior pregnancies exhibit elevated levels of health consciousness. This correlation suggests that experiences related to reproductive health may contribute to an increased awareness of overall healthcare needs and considerations.

In addition to these observations, a key insight surfaced in our study regarding the positive correlation between health consciousness and advancing age. As individuals progress through life, there appears to be a simultaneous increase in their awareness and mindfulness concerning health-related matters. This correlation underscores the evolving nature of health consciousness over the life course, with age potentially playing a role in shaping attitudes and behaviors related to health.

The scoring system applied allowed for a detailed evaluation of these aspects in the study participants. The existing literature has established a connection between certain lifestyle behaviors, such as smoking, regular alcohol consumption, or engaging in unsafe sexual practices (such as unprotected sex and frequent partner changes) and an increased risk of developing various diseases, including mouth cancer, lung cancer, sexually transmitted infections, and HPV-associated cancers [23,24]. Moderate drinking may reduce the likelihood of several diseases (diabetes, cardiovascular disease, and chronic kidney disease); however, it is associated with a two to fourfold increased risk of oral cancer and esophageal cancer [25].

Human papillomavirus (HPV) stands as the most prevalent viral infection within the reproductive tract and ranks among the most widespread causes of sexually transmitted infections globally [26]. Persistent genital high-risk human papillomavirus (HPV) infection is responsible for about 99.7% of all cervical cancer cases, which is ranked among the most prevalent cancers affecting women, with an estimated 528,000 new cases reported in 2012 alone [27]. The clinical utility of HPV testing in secondary prevention proves valuable, particularly in triaging low-grade cytological abnormalities, and exhibits greater sensitivity than cytology as a primary screening method; this is why it is increasingly becoming the primary screening method in numerous countries. As a WHO recommendation, HPV vaccination is the most cost-effective public health measure that can reduce the risk of cervical cancer [28,29].

According to the European Medicines Agency, emergency pills containing levonorgestrel are available only on prescription after consulting a practitioner in Hungary and Poland [30]. In January 2015, the European Commission issued an implementing decision allowing the sale of ECPs containing ulipristal acetate without a prescription in all EU territories.

Several countries implemented the recommendation; however its over-the-counter dispensing is still age-restricted [31]. In Hungary, all types of hormonal contraceptives, including emergency contraceptive pills, are prescription-only and require medical consultation for issuance.

Our previous study has shown that condom rupture and history of previous pregnancies are the strongest motivating factors for using emergency pills [4]. In the present study, those who were more health-conscious were significantly quicker to seek medical help compared to those with lower scores. It is known from the literature that people who practice healthy habits on a regular basis get more frequent health checkups [32]. In this study, we have described for the first time in the literature that health-conscious people seek medical help sooner.

It is important to note that the frequency of doctor visits is also affected by gender. Women are more likely to visit a doctor regularly than men [33]. In comparison to women, men in the United States are more likely to be overweight, regularly consume large amounts of alcohol, smoke more heavily and are less likely to give it up, and to use illicit drugs for non-medical purposes. Compared to women, men are less likely to use physician home visits, emergency departments, and doctor visits. In addition, compared to women, they are less likely to receive preventative care, hospice care, dental care, hospital discharges, and shorter hospital stays. Men’s shorter life expectancies may be caused by high-risk activities and low use of health services. To lessen the gender gap in public health, behavioral and preventative interventions are required [34]. In the present study, we were not able to investigate gender differences, as only women can take the post-event pill.

The study participants showed an increase in health consciousness as they grew older. Research has indicated that an individual’s frequency of general practice visits increases with age [33,35]. Compared to people under 50 (2%), men and women over 51 (27%) had screening health exams more frequently [33,35]. The discovery that 40% of women under 30 did not routinely show up for pap smears was alarming. Regarding population-based screening, information gathered by the Australian Cervical Cytology Registry indicates that over one-third of women under 30 do not show up for pap smears, and that number has been declining since 1996 [33]. Data from the United States from 1976 to 2000 further support the finding that over that time, young women’s incidence of cervical adenocarcinoma increased [36]. Delaying cervical screening raises the risk of cervical cancer spreading, according to research by Herbert et al. [33,37].

Interestingly, reproductive health awareness is also affected by previous pregnancy. In our study, the more health-conscious patients had previously been pregnant. Studies have indicated that parenthood plays a significant beneficial role in both physical and mental health, as well as self-health esteem as well [38,39,40]. Studies on the relationship between substance misuse and parenthood have shown that parents of small children smoke less frequently [41,42,43] and drink less alcohol [41,44,45]. When compared to childless persons, older parents were found to have more positive health habits, according to research by Kendig et al. on the lifestyles of older parents [41,46]. However, Becker’s work shows that, at older ages, there is no difference in lifestyle factors between childless adults and adults who have had children. It is possible that this only applies to the time that children live with their parents [41].

Our findings underscore the crucial role of health consciousness in shaping health-related behaviors and decisions. Understanding the connections between unintended pregnancy and health has the potential to contribute valuable insights to both clinical practice and health policy [47]. Individuals with heightened health consciousness exhibit healthier habits, better adherence to medical recommendations, and an enhanced quality of life [48]. Health-conscious people have a higher trust in their patient–physician relationship. Importantly, this trust was identified as mediating 28% of the overall impact of health consciousness, underscoring its considerable influence on healthcare outcomes [49]. Considering the positive correlation between health consciousness and patient–physician trust, telehealth emerges as vital in addressing the immediate healthcare needs of health-conscious individuals. The fast and accessible nature of telehealth aligns with their proactive approach, facilitating timely consultations and interventions [50].

Initiating their health education as soon as possible is crucial, fostering a heightened awareness of their well-being. This comprehensive approach not only aims to raise awareness of health considerations for the older demographic but also seeks to cultivate a heightened sense of health consciousness, especially regarding sexual health, among the younger population. By instilling this awareness early on, the goal is to empower the younger generation to effectively capitalize on modern opportunities for improved health outcomes.

### 4.1. Strengths and Limitations

This multifaceted analysis, encompassing sociodemographic details, lifestyle elements, health-related information, and an awareness score system, enhances the depth of understanding regarding the intricate relationship between lifestyle factors and reproductive health decisions. The development and application of an awareness score system provides a quantifiable measure, contributing to a structured assessment of health consciousness.

This study’s reliance on a telemedicine platform introduces the possibility of selection bias, as individuals opting for telehealth services may differ from those seeking in-person consultations. Additionally, self-reported data obtained during telehealth consultations may be susceptible to recall and social desirability biases, potentially influencing the accuracy of reported lifestyle factors. The cross-sectional design limits the establishment of causation, necessitating longitudinal studies for a more comprehensive understanding of the temporal relationships. The study primarily centers on the correlation between lifestyle factors and awareness scores with emergency contraceptive pill utilization, leaving room for further exploration of additional outcome measures in future research.

### 4.2. Implication for Practice

The observed correlation between reproductive health awareness and prompt medical consultation in emergency contraceptive use has direct implications for clinical practice. Healthcare providers should integrate discussions about lifestyle factors, such as smoking, alcohol consumption, and contraceptive practices, into routine reproductive health consultations, offering personalized interventions.

Incorporating comprehensive health education into routine care, especially focusing on reproductive health awareness, menstrual cycles, and contraceptive options, can empower patients to make well-informed decisions. The observed correlation between health awareness and prompt medical consultation underscores the value of integrating telehealth in clinical practice. Telehealth enhances accessibility, offering a convenient platform for health-conscious individuals to seek timely reproductive health consultations. This proactive approach aligns with the personalized education and counseling opportunities provided by telehealth, ensuring that individuals, regardless of location, can access quality reproductive healthcare. Embracing telehealth contributes to the prevention of unintended pregnancies by providing timely, tailored, and comprehensive reproductive health interventions.

### 4.3. Implication for Research

The identified correlation between reproductive health awareness and prompt medical consultation in emergency contraceptive use underscores critical avenues for future research in reproductive health. Investigating the dynamics of telehealth utilization in reproductive healthcare is essential. Understanding the factors influencing patients’ preferences for telehealth, potential barriers to adoption, and the impact of virtual interventions on reproductive health outcomes would contribute to a more nuanced understanding of this evolving healthcare delivery model. Conducting longitudinal studies can provide valuable insights into the sustained impact of health consciousness on reproductive health decision-making. Examining how individuals’ reproductive health awareness evolves over time and its ongoing influence on healthcare-seeking behaviors could deepen our understanding of long-term reproductive health patterns. Comparative effectiveness studies are warranted to assess the outcomes of different modes of contraceptive counseling. Comparing traditional in-person consultations with telehealth interventions can help healthcare providers determine the most efficient and patient-centered methods for delivering reproductive health information and interventions. Integrating principles of behavioral economics into research design could offer insights into the decision-making processes related to emergency contraceptive use. Understanding the behavioral factors influencing individuals’ choices and adherence to reproductive health practices can inform the development of more effective interventions. Addressing these research areas will not only deepen our understanding of the complex interplay between health consciousness, telehealth utilization, and reproductive health outcomes but also contribute to the refinement of strategies aimed at preventing unintended pregnancies and improving overall reproductive healthcare.

## 5. Conclusions

Our study highlights the significant influence of age and previous pregnancies on reproductive health awareness. More health-conscious women seek medical consultation earlier, which contributes to better health promotion and a reduced risk of health problems. Our study emphasizes the significance of lifestyle factor influence on reproductive health decisions.

## Figures and Tables

**Table 1:** Health awareness score.

Category	Points	
Smoking (0–3)	1 pack daily	0
	1 pack weekly	1
	Occasionally	2
	No smoking	3
Alcohol consumption (0–2)	Every few days	0
	Occasionally	1
	No alcohol consumption	2
Partner consistency (0–1)	None	0
	Yes	1
Pap smear in the last 2 years (0–1)	None	1
	Yes	2
Ovulation awareness (0–2)	Non-fertile period	0
	Fertile period edges days (12 or 16 days of menstrual cycle)	1
	Fertile period (13–15 days of menstrual cycle	2
Protection used (0–2)	No protection	0
	Other, e.g., withdrawal	1
	Condom	2
Previous HPV screening (0–1)	No	0
	Yes	1
Further contraception desired (0–1)	No	0
	Yes	1

**Table 2:** Patient characteristics.

**Total**	447	
Median age with 95% CI (years)	30 (29–31)	
Place of living	Countryside	Capital City
	258	189
**Category**	**Subcategory**	**Range of percent**
Smoking	1 pack daily	9.0%
	1 pack weekly	12.3%
	Occasionally	9.8%
	No smoking	68.9%
Alcohol consumption	Every few days	13%
	Occasionally	50.8%
	No alcohol consumption	36.2%
Partner consistency	None	29.5%
	Yes	70.5%
Pap smear in the last 2 years	None	33.6%
	Yes	66.4%
Ovulation awareness	Non-fertile period	70.9%
	Fertile period edges days (12 or 16 days of menstrual cycle)	9.0%
	Fertile period (13–15 days of menstrual cycle	20.1%
Protection used	No protection	29.8%
	Other, e.g., withdrawal	10.3%
	Condom	59.9%
Previous HPV screening	No	84.6%
	Yes	15.4%
Further contraception desired	No	67.1%
	Yes	32.9%

Categorical parameters are presented as n. Continuous data are presented as median with interquartile range.

## Data Availability

The published article contains all generated and analyzed data from this series.
